# Use of Rapamycin in a Patient With Juvenile Myelomonocytic Leukemia: A Case Report

**DOI:** 10.1177/2324709617728528

**Published:** 2017-09-08

**Authors:** Shivani Y. Upadhyay, Satiro N. De Oliveira, Theodore B. Moore

**Affiliations:** 1 University of California, Los Angeles, CA, USA; 2 Cedars Sinai Medical Center, Los Angeles, CA, USA

**Keywords:** JMML, graft-versus-host disease, rapamycin, transplantation

## Abstract

The relapse rate for children with juvenile myelomonocytic leukemia (JMML) status post hematopoietic stem cell transplantation (HSCT) approaches 50% within 5 years. Graft-versus-leukemia (GVL) is thought to play important role in the treatment of JMML. For this reason, careful management of immunosuppressive drugs after HSCT is crucial. This case report demonstrates that rapamycin and GVL represent a viable medical strategy for the management of pediatric patients with JMML who relapse following status post-HSCT.

## Introduction

Juvenile myelomonocytic leukemia (JMML) is a rare myeloproliferative disorder that occurs in early childhood, often characterized by peripheral blood monocytosis, monosomy 7, dysplastic bone marrow, granulocyte-macrophage colony-stimulating factor (GM-CSF) hypersensitivity, and increased fetal hemoglobin.^1,2^ The standard treatment for JMML is chemotherapy followed by hematopoietic stem cell transplantation (HSCT), but the relapse rate for children with JMML status post-HSCT approaches 50% within 5 years^1,3,4^ and remains the main cause of therapy failure and mortality.

Progress has been made in understanding the molecular genetics and mechanisms of this disorder, implicating hyperactive RAS as essential initiating event.^5^ Hyperactive RAS mutation also has an effect on the PI3K/Akt/mTOR pathway, which is involved in numerous cellular processes such as cell growth, survival, and death.^6-8^ Rapamycin, a commonly used immunosuppressant, inhibits the mTOR pathway, and it is currently being used after HSCT for prevention of graft-versus-host disease (GVHD). In addition, preclinical evidence suggests a potential therapeutic role for mTOR inhibition in JMML.^9^ In this case report, we describe an infant with JMML status post-HSCT with evidence of graft loss and disease recurrence, but currently doing well following withdrawal of immunosuppression and initiation of rapamycin.

## Case Report

A 7-month-old Hispanic female presenting initially with anemia, thrombocytopenia, elevated white cell count, and splenomegaly.^10^ The results of the bone marrow biopsy were as follows: negative monosomy 5 or 7, trisomy 8, 9:22 translocation or neurofibromatosis type 1 gene deletion. Studies confirmed GM-CSF hypersensitivity and N-RAS mutation. The bone marrow was also negative for partial deletions of the long arms of chromosomes 5, 7, and 20. The patient met diagnostic criteria for JMML. She was initially treated with intensive chemotherapy that consisted of fludarabine 30 mg/m^2^/day × 5 days, cytarabine 2 g/m^2^/day × 5 days, and isotretinoin 100 mg/m^2^/day continuously. She tolerated cycles 1 and 2 of chemotherapy relatively well; however, isotretinoin had to be held during the first cycle secondary to severe skin rash. The patient responded to chemotherapy, and the liver and spleen decreased significantly in size. She then received 2 more cycles of chemotherapy in preparation for progression into HSCT 4 months from diagnosis, with bone marrow remission and no hepatosplenomegaly. Due to the high risk of relapse, our group decided to use a radiation-containing regimen despite the patient’s age, with the addition of ATG for an umbilical cord blood graft. The patient was conditioned with total body irradiation (1200 Gy fractioned into 8 doses from day −7 to day −4), cyclophosphamide 60 mg/kg/day × 2 days (days −3 and −2), rabbit ATG (Thymoglobulin, Genzyme) 3 mg/kg/day × 2 days (days −2 and −1), and then received a 5/6 unrelated umbilical cord transplant (A locus mismatch).^11,12^ The cord blood total nucleated cell (TNC) was 89.38 × 10^7^ in 55 mL, resulting in TNC dose of 9.6 × 10^7^ TNC/kg and CD34+ cell dose of 6.47 × 10^5^ cells/kg. Neutrophils were engrafted on day +15, the patient was discharged on day +22, and platelet engraftment was documented on day +36. She was initially started on cyclosporine (intravenously continuous at 5 mg/kg/day on day −2) and methylprednisolone (1 mg/kg/day starting on day +3) for GVHD prophylaxis. After transplantation, her spleen dimensions returned to appropriate size for age and she had full donor engraftment, as determined by restriction fragment length polymorphism of cellular DNA (RFLP). Immunosuppression was prolonged due to the development of incipient hemolytic anemia due to HSCT blood mismatch (patient was O Rh positive and the umbilical cord blood was A Rh positive). At 9 months posttransplantation, she was diagnosed with relapsed JMML, presenting hepatosplenomegaly and fevers. RFLP studies in the bone marrow demonstrated less than 20% donor DNA. Her immunosuppression drugs were completely withdrawn and she developed grade III GVHD. The maximum manifestations of acute GVHD were stage 3 skin, stage 2 liver, stage 0 gastrointestinal, and chronic GVHD was extensive skin rash and abnormal LFTs with confirmatory biopsy.

The patient’s GVHD was successfully controlled by initiating rapamycin at the dose of 0.01 mg/kg. Now about 77 months later, this patient remains on rapamycin at low dose (0.14 mg/m^2^/day) and her malignancy is in remission, with stable donor engraftment in peripheral blood (68% donor DNA at 3 years after relapse, 77% donor DNA at 5 years after relapse), and complete resolution of her organomegaly. Over the last year, her blood counts were white blood cells 4.8 to 9.5 × 10^3^/µL, hemoglobin 11.4 to 12 g/dL, platelets 280 to 420 × 10^3^/µL, and absolute neutrophil count 1.8 to 5.4 × 10^3^/µL, never showing any blasts or dysplastic cells.

## Discussion and Conclusion

JMML is a rare malignancy that makes up less than 1% of all childhood leukemias.^13^ Currently, there is no consistently effective treatment of JMML. Chemotherapy alone is rarely sufficient to cure these patients, and HSCT has only been shown to extend survival.^3,14-16^ The relapse rate for children with JMML status posttransplantation remains as high as 50% within 5 years.^1,3,17^ For this reason, attempts have been made to optimize the transplantation conditioning regimens to improve survival. The other transplantation variable that may impact event-free survival is GVL.^18,19^ The potential impact of GVHD on relapse has previously been described for many types of leukemia including some patients with JMML.^2,20-22^ A case by Tanoshima et al^21^ reported a 13-month-old female who received an unrelated cord blood transplant for JMML. The patient had disease progression even after the conditioning regimen, which consisted of busulfan, fludarabine, and melphalan. However, complete remission was achieved with the development of GVHD and decreased immunosuppression. The case shows that GVL can be effective against JMML. A report from Orchard et al^22^ described a 4-year-old girl who relapsed early after an unrelated donor HSCT. There was evidence of blast cells and a decrease in donor DNA by polymerase chain reaction–based VNTR (variable nucleotide tandem repeat). Immunosuppression was then withdrawn, and the patient then developed GVHD. Repeat peripheral blood and marrow VNTR testing then showed an increase in donor DNA. Another report^20^ described treating a relapsed JMML patient after HSCT by using donor lymphocyte infusions. All these cases demonstrate that GVL may affect JMML. Immediate discontinuation of GVHD prophylaxis in patients with disease recurrence after allogeneic HSCT may lead to remission of malignancy, but can lead to chronic GVHD.^1^

Targeted therapy may also play an important role in preventing disease progression. Searching for compounds that would be able to inhibit various components of the signaling transduction pathways in JMML has been an area of significant interest.^23^ A known pathway that is involved in JMML and has been studied is the RAS pathway. RAS mutations have been known to activate PI3/Akt/mTOR. This PI3/Akt/mTOR pathway can be inhibited by rapamycin.^8^ Rapamycin can bind to the binding protein FK506 and FKBP-12, which can then bind to and inhibit mTOR. Rapamycin is a US Food and Drug Administration–approved drug that is being used as an immunosuppressant in solid organ transplants as well as HSCT to prevent GVHD. Because rapamycin causes mTOR inhibition it may also provide an antileukemic effect as well as serve as an immunomodulator.

Patients with JMML who relapse following HSCT may benefit from interventions that may include targeted therapies and careful immunomodulation, thus maximizing the GVL effect, as demonstrated in our case report.
